# Molecular docking analysis of penta-galloyl-glucose with VEGF signaling molecules

**DOI:** 10.6026/97320630017924

**Published:** 2021-11-30

**Authors:** Karthick Dharmalingam, Velvizhi Dharmalingam, Satheesh Durairaj, Praveen Sharma, Selvaraj Jayaraman

**Affiliations:** 1 Department of Biochemistry, All India Institute of Medical Sciences, Jodhpur, Rajasthan - 342005, India; 2 Department of Chemistry, Captain Srinivasa Murthy Central Ayurveda Research institute, CCRAS, Ministry of AYUSH, Government of India, Chennai - 600106; 3 Department of chemistry, Siddha Central Research institute, Arumbakkam, Chennai - 600106; 4 Department of Biochemistry, Saveetha Dental College and Hospitals, Saveetha Institute of Medical and Technical Sciences, Saveetha University, Chennai-600 077, India

**Keywords:** Angiogenesis, VEGF signaling, penta galloyl glucose, molecular docking

## Abstract

It is of interest to document the molecular docking analysis data of penta-galloyl-glucose with VEGF signaling molecules in the context of cancer. Data shows that penta-galloyl-glucose have optimal binding affinities with VEGF-A,VEGFR-2, PKC, RAF, MEK, ERK and AKT with binding affinity of -7.9,-8.3,-8.6, -3.7,10.1,-9 and -10.8 kcal/mol respectively for further consideration in this context.

## Background:

Drug discovery for the treatment of cancer is gaining momentum in recent years [1]. The most common process is angiogenesis, which involves the development of new blood vessels from pre-existing ones [2]. Most anti-angiogenic treatments target endothelial cells with the formation of new blood vessels [3]. Novel therapeutic targets for anti-angiogenic drugs must be identified, and treatments tailored to each patient must be proposed [4]. VEGF signaling molecules including as VEGF-A, VEGFR-2, PKC, RAF, MEK, ERK, and AKT have emerged as a promising therapeutic target for the development of new anti-angiogenic drugs. Hence, it is necessary to understand its molecular interactions using emerging tools [5-6]. Therefore, it is of interest to document the molecular docking analysis of penta-galloyl-glucose with VEGF signaling molecules such as VEGF-A,VEGFR-2, PKC, RAF, MEK, ERK and AKT.

## Materials & Methods:

### Protein preparation:

3D structures of VEGF-A, VEGFR-2, PKC, RAF, MEK, ERK and AKT were downloaded from the RCSB/PDB database using PDB IDs (IBJ1; 1Y6A; 2I0E; 3OMV; 3E8N; 1TVO and 3O96). Water molecules and all non-standard residues have been eliminated from the basic structure using AutoDock. Missing hydrogens and charges were added and the protein receptor was saved as a pdbqt file into the PyRx's workspace folders [7].

### Preparation of ligand:

The penta-galloyl-glucose structure was downloaded from the Pubchem database in 2D SDF format. The compounds were translated to 3D PDB format using the online smiles translator. The structures of the compounds were then loaded into PyRx and made into a ligand pdbqt file by clicking Load Molecule and create as ligand pdbqt file [8].

### Molecular docking:

AutoDock in PyRx PyRx (0.8) GUI was used to compute the binding capability of the selected compound with each of the selected targets. Docking provides valuable insights into the interaction between the proteins and ligand. The structures of the compound were prepared before docking using the make ligand command present in PyRx. Make macromolecule option was used to prepare the protein structures. AutoDock program along with the Lamarckian genetic algorithms (LGA) was used for docking. The Lamarckian GA parameters used in the analysis consist of 30 independent runs, 150 size of population, a 25,000,000 energy evaluations, 27,000 number of generation, mutation rate of 0.02, and 0.8 crossover rate. Docking was carried out within the pre-defined grid size in proteins. Individual docking procedures was performed for each ligand protein complex. The results were ranked in the order of increasing docking energies. The lowest binding energy of each cluster was assumed to be representative [9,10].

## Results and Discussion:

A study on the anti angiogenic effect of penta-galloyl-glucose using molecular docking with VEGF signaling molecules is of interest. The effect of the penta-galloyl-glucose formulation on various protein components of the VEGF signaling cascade is one of the primary routes controlling angiogenesis. AutoDock -PyRx was used for molecular docking. The data were examined based on the ligand's top 10 conformations with the lowest binding energy. PyMol was used to show the H-bond interaction. Table 1(see PDF) shows the results of docking penta-galloyl-glucose against these protein targets. Penta-galloyl-glucose showed highest binding affinity to most of the targets (VEGF-A, VEGFR-2, PKC, RAF, MEK, ERK and AKT). Results showed that penta-galloyl-glucose showed the strong interaction with most of the selected target with good binding energies ranging from -3.7 to 10.8 kcal/mol. The binding energies, in combination with the interaction profile (hydrogen bond interactions), give a good indicator of the ligand's affinity and stability with the protein. All of the selected target proteins produced a large number of hydrogen bond interactions with penta-galloyl-glucose. Figure 1 and Table 1 show the hydrogen bond interactions established between proteins and penta-galloyl-glucose. Penta-galloyl-glucose has a high affinity for the key proteins involved in VEGF signaling for further consideration in drug discovery for cancer.

## Conclusion:

We document the molecular docking analysis data of Pentagalloylglucose with VEGF signaling molecules such as VEGF-A,VEGFR-2, PKC,RAF, MEK,ERK, and AKT for further consideration in this context.
